# Pathological Regression of Lymph Nodes Better Predicts Long-term Survival in Esophageal Cancer Patients Undergoing Neoadjuvant Chemotherapy Followed by Surgery

**DOI:** 10.1097/SLA.0000000000004238

**Published:** 2020-07-14

**Authors:** Takaomi Hagi, Tomoki Makino, Makoto Yamasaki, Kotaro Yamashita, Koji Tanaka, Takuro Saito, Tsuyoshi Takahashi, Yukinori Kurokawa, Masaaki Motoori, Yutaka Kimura, Kiyokazu Nakajima, Eiichi Morii, Hidetoshi Eguchi, Yuichiro Doki

**Affiliations:** ∗ Department of Gastroenterological Surgery, Osaka University Graduate School of Medicine, Osaka, Japan; † Department of Surgery, Osaka General Medical Center, Osaka, Japan; ‡ Department of Surgery, Faculty of Medicine, Kindai University, Osaka, Japan; § Department of Pathology, Graduate School of Medicine, Osaka University, Osaka, Japan.

**Keywords:** esophageal cancer, lymph node, neoadjuvant chemotherapy, pathological response

## Abstract

To evaluate pathological response to NAC in metastatic LNs, and assess its clinical prognostic significance in patients with EC.

**Summary of Background Data:**

The pathological response to preoperative treatment is commonly evaluated in the PT. However, LN metastases strongly correlate with systemic micro-metastases. Thus, pathological evaluation of LN response could more accurately predict prognosis in EC patients undergoing NAC before surgery.

**Methods:**

We enrolled 371 consecutive patients who underwent triplet NAC followed by surgery for EC between January 2010 and December 2016. Pathological LN regression grade was defined by the proportion of viable tumor area within the whole tumor bed area for all metastatic LNs: grade I, >50%; II, 10%–50%; III, <10%; and IV, 0%. We analyzed the correlation of grade with clinico-pathological parameters.

**Results:**

Among 319 patients with clinically positive LNs, pathological LN regression grades were I/II/III/IV in 115/51/58/95 patients, and 191 patients (59.9%) showed discordance between the PT and LN pathological regression grades. LN regression grade significantly correlated with cN positive number, ypTNM, lymphovascular invasion, and clinical/pathological PT response. Multivariate analysis for recurrence-free survival revealed that LN regression grade [hazard ratio (HR) = 2.25, *P <* 0.001], ypT (HR = 1.65, *P =* 0.005), and ypT (HR = 1.62, *P =* 0.004) were independent prognostic factors, but not pathological PT regression grade *(P =* 0.67).

**Conclusions:**

Compared to PT response, pathological LN response better predicted long-term survival in EC patients who received NAC plus curative surgery.

Esophageal cancer (EC) is a common digestive tract malignancy, and the sixth leading cause of cancer death worldwide.^1^ Neo-adjuvant chemotherapy (NAC) has recently become a standard treatment for locally advanced EC, based on the results of several randomized control trials.^2^^,^^3^ NAC may induce tumor down-staging, increased resectability, and elimination of micrometastases, thus leading to survival benefits for patients.

Clinical or pathological responses are routinely evaluated to help predict prognosis in patients who undergo NAC for EC.^4^ The commonly used grading systems for pathological tumor regression refer to the amount of therapy-induced fibrosis relative to residual tumor (Mandard system)^5^ or to the estimated percentage residual tumor relative to the previous tumor site (Becker system).^6^^,^^7^ These systems are generally considered good indicators that provide important prognostic information or estimated risk of disease recurrence; however, they only reflect the therapeutic effect on the primary tumor (PT). To date, no standard grading systems evaluate the therapeutic response in lymph nodes (LNs).

Evidence shows that compared to PT progression, LN metas-tases in EC patients are strongly associated with poor prognosis.^8^,^9^,^10^ Moreover, in recent studies, 18-fluorodeoxyglucose positron emission tomography reveals inconsistent responses to NAC between PT and LNs,^11^^,^^12^ implying additional value of evaluating the clinical response in LNs. We hypothesized that in patients who have undergone NAC for EC, the pathological LN response might be a better prognostic factor than the pathological PT response. In our previous evaluation of tumor diameter using computed tomography (CT), we demonstrated that clinical LN response to NAC was a better prognostic factor for long-term survival than the PT response.^13^ However, scarce data are available regarding pathological evidence of LN responses to NAC and the clinical significance.

In the present study, we aimed to investigate a new pathological grading system that reflects the LN response to NAC. We evaluated the use of this system for predicting long-term survival in patients who underwent curative surgery following NAC for locally advanced EC.

## METHODS

### Patients

This retrospective study included consecutive patients who underwent triplet NAC—with either Adriamycin, cisplatin, and 5-fluorouracil (ACF) or docetaxel, cisplatin, and 5-fluorouracil (DCF)—followed by surgery for EC, between January 2010 and December 2016, in the Department of Gastroenterological Surgery, Osaka University Hospital.^14^,^15^,^16^,^17^ The selection of NAC for patients with EC was based on the TNM classification,^18^^,^^19^ with cT1–3N1–3 disease being a definite indication, and either cT3N0 or cT4Nany, excluding massive infiltration to the bronchus or aorta considered a relative indication. Massive invasion to adjacent organs was an indication for chemoradiotherapy (CRT). Patients with cT1–2N0 tumors underwent surgery with no preoperative therapy. The eligibility criteria were histological diagnosis with squamous cell carcinoma of the esophagus, including the esophagogastric junction but excluding the cervical esophagus; absence of distant metastasis, excluding nonregional LN metastases; and having undergone mac-roscopically curative resection (R0/1). Among 539 EC patients who received preoperative therapy followed by surgery, we excluded 79 who received a NAC regimen other than ACF or DCF, and 66 who underwent CRT. Of the remaining 394 patients, 371 patients achieved curative resection meeting the eligibility criteria of this study (Supplementary Fig. S1, http://links.lww.com/SLA/C378). True cN negative cases, defined as negative LNs with no evidence of regression in pathological examination, were excluded from the analysis of relationships between LN regression grade and clinicopathological characteristics or survival.

Clinical response of the PT was assessed following the Japanese Classification of Esophageal Cancer.^20^ Patients with complete or partial response were considered responders, whereas those with stable or progressive disease were considered nonresponders. To calculate the sum of LN minor axes, each LN minor axis was measured using enhanced 64-slice CT scanning before NAC, as previously described.^13^ All patients were staged according to the eighth edition of the Union for International Cancer Control TNM classification and staging system.^21^ Clinical staging was performed based on the pre-treatment findings of therapeutic endoscopy, CT scan, and 18-fluorodeoxyglucose positron emission tomography. LNs with a short diameter of ≤10 mm and standard uptake values (SUV)max of ≤2.5 were considered clinically negative (ie, cN0), as previously described.^13^ Each included patient provided signed consent, and this study was approved by the Institutional Review Board of Osaka University Hospital.

### Treatment of EC

The standard NAC regimen comprises 2 or 3 courses of triplet chemotherapy: ACF or DCF.^22^^,^^23^ The ACF regimen involved intravenous Adriamycin (35 mg/m^2^) and cisplatin (70 mg/m^2^) on day 1, and continuous intravenous infusion of 5-fluorouracil (700 mg/m^2^) on days 1–7, repeated every 4 weeks. The DCF regimen included intravenous docetaxel (70 mg/m^2^) and cisplatin (70 mg/m^2^) on day 1, and continuous intravenous infusion of 5-fluorouracil (700 mg/m^2^) on days 1–5, repeated every 3 weeks. After completion of the last course of chemotherapy, the patients underwent surgery. The standard surgical procedure was subtotal esophagectomy with 2-field or 3-field lymphadenectomies, as defined in the Japanese Classification of Esophageal Cancer.^18^^,^^24^^,^^25^ The present study included patients who received a reduced dose of each agent due to severe toxicity, and those who did not respond to NAC.

### Pathological Examination of PT and LN Regression

Pathological examination of resected specimens was initially performed following a protocol for pathological examination, and in accordance with the Japanese Classification of Esophageal Can-cer.^18^^,^^26^ Briefly, after formalin fixation, the PT was cut into 5-mm slices parallel with the long axis of the esophagus. LNs were typically divided into 2 pieces, generating the maximum sectioned surface. Section slides were stained with hematoxylin and eosin, and carefully examined by an experienced pathologist (E.M.). Pathological PT regression was graded into 4 categories based on residual tumor pertumorbed (gradeI, >50%; gradeII, 10%–50%; gradeIII, <10%; grade IV, 0%) as originally described by Becker et al.^7^^,^^8^ Patients with PT regression grade of III–IV were classified as PT responders, and those with grades of I–II as PT nonresponders.

**FIGURE 1:**
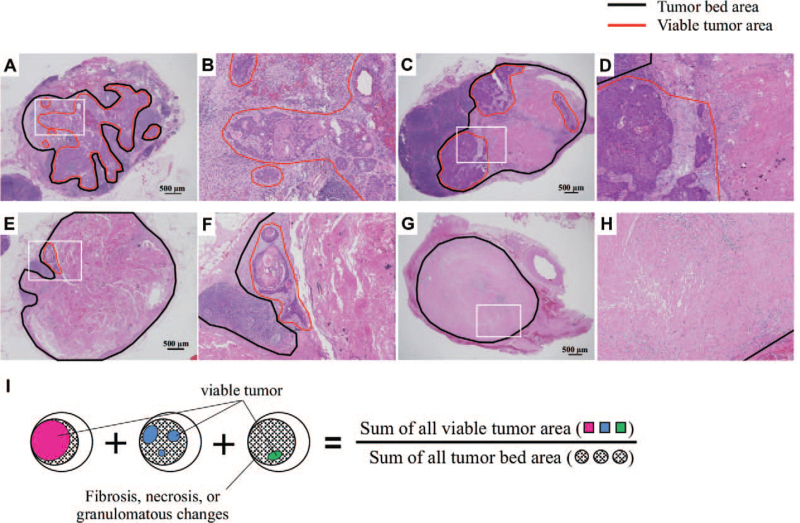
Representative images of hematoxylin and eosin-stained lymph nodes (LNs) for each LN regression grade. A, Grade I: Viable tumor cells are observed in over 50% of the tumor bed area. B, Higher magnification of the white squared area of panel A. C, Grade II: Viable tumor cells are observed in 10%–50% of the tumor bed area. D, Higher magnification of the white squared area of panel C. E, Grade III: Viable tumor cells are observed in <10% of the tumor bed area. F, Higher magnification of the white squared area of panel E. G, Grade IV: No viable tumor cells observed in the tumor bed area. H, Higher magnification of the white squared area of panel G. The area enclosed by black lines indicates the tumor bed area, and the area enclosed by red lines indicates the viable tumor area. I, Schematic representation of the formula for calculating the total LN regression grade.

The therapeutic effect of NAC on LNs was assessed based on a substantial area of fibrosis, necrosis, and granulomatous changes within the nodal parenchyma, as previously described.^27^,^28^,^29^,^30^ LN regression grade was determined by calculating the proportion of viable tumor area within the whole tumor bed area (including the area of viable tumor and therapeutic changes of NAC) within the LN (Fig. 1A–H). For patients with multiple metastatic LNs (including LNs with complete response), we generated a total LN regression grade based on the proportion of the summed viable tumor area relative to the summed tumor bed area for all metastatic LNs (Fig. LN regression grades were determined using the same criteria as for PT regression grades. This system was previously validated in a subset of 30 patients using NIH ImageJ software (Bethesda, MD) to calculate the areas of each LN photographed using a BZ-X710 microscope (KEY-ENCE; Itasca, IL). We additionally assessed the software quality by using it to grading cases with 6 or more metastatic LNs. Cases negative for LN metastasis, and with no evidence of regression or previous tumor involvement, were recorded as “true cN negative.”

### Statistical Analysis

We analyzed the relationships between clinicopathological characteristics and LN regression grades using the chi-square test for categorical variables and Mann-Whitney *U* test for continuous variables. Recurrence-free survival (RFS) was defined as the interval from the date of surgery to the date of recurrence or death from any cause. Cumulative recurrence was defined as the interval from the date of surgery to the date of recurrence. RFS and cumulative recurrence were estimated using the Kaplan-Meier method, and compared using the log-rank test. A Cox proportional hazard model was used for univariate analyses of RFS, and the prognostic variables that showed significant association were further assessed by multivariate analyses. *P <* 0.05 was considered to indicate statistical significance. All analyses were performed using SPSS software, version 22.0 (IBM Corp., Armonk, NY).

## RESULTS

### Patient Characteristics and Distribution of LN Regression Grade

Among 371 eligible patients, 52 had negative LNs with no evidence of regression (true cN negative group). The remaining 319 patients were evaluated for LN regression grade. Supplementary Table S1, http://links.lww.com/SLA/C381 summarizes the baseline characteristics of all 371 patients. The median age was 68 years (range, 35–83 years) and the patients were predominantly male (85.7%). The median number of clinically positive LNs was 2 (range, 0–41) and the distribution of cStage was 26 stage I, 97 stage II, 182 stage III, and 66 stage IV. NAC was performed using the ACF regimen in 107 patients (28.8%), and the DCF regimen in 264 patients (71.2%).

**FIGURE 2:**
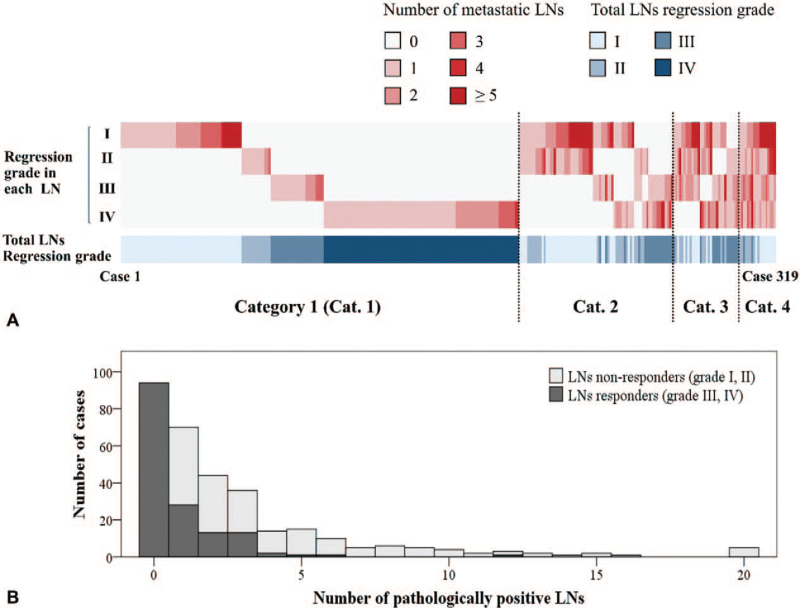
A, Distribution of the lymph node (LN) regression grades in each patient, and of the total LN regression grade among 319 cases with clinically positive LNs. Shades of red are used to map the number of metastatic LNs in each regression grade, and shades of blue map the total LN regression grades. Dotted lines distinguish cases by the number of different regression grades exhibited by LNs in a single case. Cat. indicates category. B, Bar chart showing the number of patients with LNs responders and nonresponders by each number of pathologically positive LNs among 319 cases with clinically positive LNs. The white area represents the number of patients with nonresponders and the dark gray area represents the number of patients with responders.

Total LN regression grade was I in 115 patients (36.1%), II in 51 patients (16.0%), III in 58 patients (18.2%), and IV in 95 patients (30.0%) (Table 1). Figure 2A shows the distribution of LN regression grade within each patient. Concerning the distribution of regression grades for each LN per patient, 194 patients (60.8%) exhibited no heterogeneity regarding their LN responses (category 1), 75 (23.5%) patients showed LN responses of 2 different grades (category 2), 32 (10.0%) patients showed LN responses of 3 different grades (category 3), and 18 (5.6%) exhibited LN responses of 4 different grades (category 4). Table 1 shows the relationship between the PT regression grade and the LN regression grade. Of 319 patients, 128 (40.1%) showed concordance between the PT and LN regression grade, 153 patients (48.0%) showed better response in LNs compared to PT, and 38 (11.9%) showed better response in PT than in LNs.

**TABLE 1:** Correlation Between Primary Tumor (PT) and Lymph Node (LN) Pathological Regression Grades

		PT Regression Grade				
		I	II	III	IV	Total
Total LN regression grade	I	89	15^†^	8^†^	3^†^	115 (36.1%)
	II	**38** ^∗^	6	5^†^	2^†^	51 (16.0%)
	III	35^∗^	8^∗^	**10**	5^†^	58 (18.2%)
	IV	37^∗^	18^∗^	17^∗^	**23**	95 (30.0%)
	Total	199 (62.4%)	47 (14.7%)	40 (12.5%)	33 (10.3%)	319 (100%)

Bold numbers surrounded with bold squares indicate patients in whom the total LN regression grade coincided with the PT regression grade. Shaded cells indicate “responders” in terms of PT or total LN regression grade.

∗ Patients in whom the total LN regression grade was better than the PT regression grade.

† Patients in whom the total LN regression grade was worse than the PT regression grade.

### Relationship Between Clinico-pathological Parameters and Pathological Regression Grade of LNs

Table 2 shows the relationship between clinicopathological characteristics and LN responses according to total LN regression grade. Compared with nonresponders, patients who showed a response in terms of total LN regression grade included significantly higher proportions of ypT0-2 (60.1% vs 42.2%, *P =* 0.001), ypN0-1 (88.2% vs 44.0%, *P* < 0.001), ypM0 (95.4% vs 80.7%, *P* < 0.001), negative lymphatic invasion (46.9% vs 17.2%, *P* < 0.001), negative vascular invasion (74.1% vs 58.6%, *P* = 0.005), clinical PT response (75.2% vs 63.9%, *P* = 0.029), and responders in terms of pathological PT regression grade (35.9% vs 10.8%, *P* < 0.001). Compared to nonresponders, responders in terms of total LN regression grade exhibited significantly lower numbers of cN-positive LNs (median: 3 vs 1, *P* < 0.001) The sum of LN minor axes, as measured by pre-therapeutic CT scan, did not significantly differ among the 4 groups with different total LN regression grades (Supplementary Fig. S2, http://links.lww.com/SLA/C379). Similarly, compared to nonres-ponders, responders in terms of total LN regression grade showed significantly fewer pN-positive LNs (median of 3 vs 0, *P <* 0.001). Most responders (96.7%, 148/153) had ≤3 pN-positive LNs, as shown in Figure 2B.

**TABLE 2:** Clinicopathological Characteristics According to Total Lymph Node (LN) Regression Status

	Responders^∗^ (n = 153, %)	Nonresponders^†^ (n = 166, %)	*P*-value
Age in years			
Median (range)	69 (35–82)	66 (38–82)	0.071
Sex			
Male	134 (87.6%)	140 (84.3%)	0.41
Female	19 (12.4%)	26 (15.7%)	
Location			
Ut	26 (17.0%)	34 (20.5%)	0.43
Mt/Lt	127 (83.0%)	132 (79.5%)	
Histological differentiation (SCC)			
Well/mod	137 (89.5%)	148 (89.2%)	0.91
Poor	16 (10.5%)	18 (10.8%)	
cT			
1–2	37 (24.2%)	33 (19.9%)	0.35
3–4	116 (75.8%)	133 (80.1%)	
cN			
0–1	136 (88.9%)	140 (84.3%)	0.23
2–3	17 (11.1%)	26 (15.7%)	
cM			
0	126 (82.4%)	128 (77.1%)	0.25
1	27 (17.6%)	38 (22.9%)	
Number of cN			
Median (Range)	1 (1–18)	3 (1–41)	**<0.001**
Summed minor axes of pretreatment LNs (mm)			
Median	22.9	24.2	0.23
Range	8.3–115.5	7.5–124.7	
NAC regimen			
ACF	40 (26.1%)	56 (33.7%)	0.14
DCF	113 (73.9%)	110 (66.3%)	
Number of dissected LNs			
Median (range)	56 (14–154)	61 (21–185)	0.053
0–2	92 (60.1%)	70 (42.2%)	0.001
3–4	61 (39.9%)	96 (57.8%)	
ypN			
0–1	135 (88.2%)	73 (44.0%)	**<0.001**
2–3	18 (11.8%)	93 (56.0%)	
Number of pN			
Median (range)	0 (0–12)	3 (1–40)	**<0.001**
ypM			
0	146 (95.4%)	134 (80.7%)	**<0.001**
1	7 (4.6%)	32 (19.3%)	
Lymphatic invasion^‡^			
Negative	67 (46.9%)	27 (17.2%)	**<0.001**
Positive	76 (53.1%)	130 (82.8%)	
Vascular invasion^‡^			
Negative	106 (74.1%)	92 (58.6%)	**0.005**
Positive Clinical response of PT^§^	37 (25.9%)	65 (41.4%)	
Responder	115 (75.2%)	106 (63.9%)	**0.029**
Nonresponder Pathological PT regression grade^||^	38 (24.8%)	60 (36.1%)	
Responder	55 (35.9%)	18 (10.8%)	**<0.001**
Nonresponder	98 (64.1%)	148 (89.2%)	

∗ Defined as total LN regression grade of III or IV.

† Defined as total LN regression grade of I or II.

‡ Unknown in 19 patients.

§ Responder was defined as complete or partial response, and nonresponder as stable or progressive disease.

|| Responder was defined as grade III or IV, and nonresponder as grade I or II. ACF indicates adriamycin, cisplatin, and 5-fluorouracil; DCF, docetaxel, cisplatin, and 5-fluorouracil; Lt, lower thoracic esophagus including esophagogastric junction; Mt, middle thoracic esophagus; NAC, neoadjuvant chemotherapy; PT, primary tumor; SCC, squamous cell carcinoma; Ut, upper thoracic esophagus.

### Relationship Between Long-term Survival and Pathological Regression Grade of LNs

**FIGURE 3:**
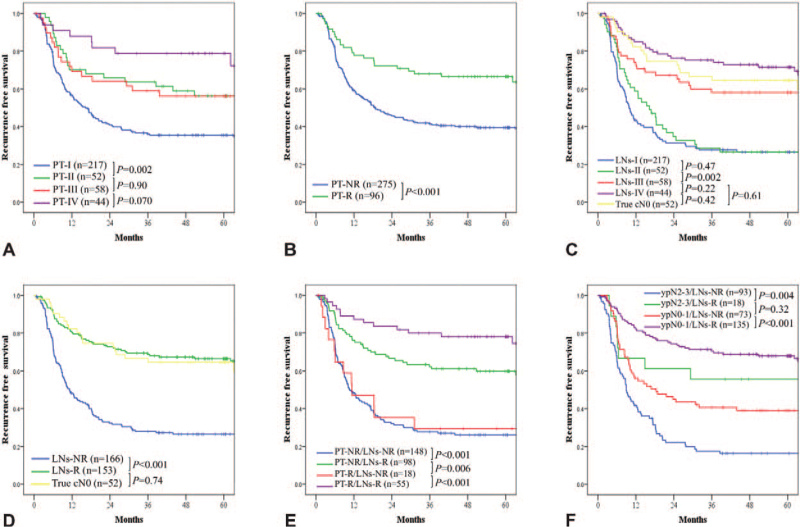
Kaplan-Meier recurrence-free survival (RFS) for all 371 patients according to (A) the primary tumor (PT) regression grade, (B) nonresponders and responders in terms of PT regression grade, (C) total lymph node (LN) regression grade, and (D) nonresponders and responders in terms of total LN regression grade. (E) Kaplan-Meier RFS for 319 patients with clinically positive LNs classified into 4 groups based on total LN regression grade along with PT regression grade. NR indicates nonresponders;R, responders.

The median follow-up RFS was 60.6 months among censored patients. The median RFS for all patients was 36.1 months, with a 95% confidence interval (CI) of 11.6–60.5 months, and 194 events (52.3%) were identified. The 5-year RFS rates for patients with PT regression grades of I, II, III, and IV, were 36.9%, 59.2%, 58.4%, and 77.3%, respectively (Fig. 3A). Compared to nonresponders, responders in terms of PT regression grade showed a significantly higher 5-year RFS rate (41.7% vs 67.1%, *P* < 0.001, Fig. 3B).

The 5-year RFS rates for patients with LN regression grades of I, II, III, IV, and true cN0 were 26.5%, 26.6%, 58.1%, 71.5%, and 64.5%, respectively (Fig. 3C). RFS in true cN0 patients was significantly better than in patients with a total LN regression grade of I (*P* < 0.001) or II (*P* < 0.001), but not compared to patients with a total LN regression grade of III (*P* = 0.61) or IV (*P* = 0.42). The 5-year RFS rate among responders in terms of LN regression grade was significantly better than that of nonresponders (66.4% vs 26.5%, *P* < 0.001) and almost equivalent to that in the true cN0 group (66.4% vs 64.5%, *P* = 0.74, Fig. 3D). The 5 clinico-pathological covariables that exhibited statistical significance in univariate analysis and were included in Cox multivariate analysis for RFS. Poor RFS was independently predicted by ypT3-4 [hazard ratio (HR) = 1.65; 95% CI, 1.16–2.33; *P* = 0.005], ypN2-3 (HR = 1.62; 95% CI, 1.16–2.26; *P* = 0.004), and nonresponse in terms of total LN regression grade (HR = 2.25; 95% CI, 1.56–3.24; *P* < 0.001), but not by nonresponse in terms of pathological PT regression grade (HR = 1.10; 95% CI, 0.69–1.76; *P* = 0.67) (Table 3).

**TABLE 3:** Univariate and Multivariate Analysis of Recurrence-free Survival

	Univariate Analysis		Multivariate Analysis	
	HR (95% CI)	*P*-value	HR (95% CI)	*P*-value
Age (yr)				
<65	1.13 (0.83–1.54)	0.44		
≥65	1			
Sex				
Male	1.01 (0.65–1.57)	0.95		
Female	1			
Location				
Ut	1	0.97		
Mt/Lt	1.01 (0.69–1.47)			
Histological differentiation (SCC)				
Well/mod	1			
Poor	1.21 (0.77–1.92)	0.41		
NAC regimen				
ACF	1.04 (0.75–1.43)	0.83		
DCF	1			
ypT				
0–2	1	**<0.001**	1	**0.005**
3–4	2.20 (1.62–2.99)		1.65 (1.16–2.33)	
ypN				
0–1	1	**<0.001**	1	**0.004**
2–3	2.62 (1.94–3.54)		1.62 (1.16–2.26)	
Clinical response of PT^∗^				
Responders	1	**0.006**	1	0.54
Nonresponders	1.55 (1.14–2.11)		1.11 (0.79–1.56)	
Pathological PT regression grade^†^				
Responders	1	**<0.001**	1	0.67
Nonresponders	2.12 (1.40–3.23)		1.10 (0.69–1.76)	
Total LN regression grade^†^				
Responders	1	**<0.001**	1	**<0.001**
Nonresponders	3.04 (2.20–4.20)		2.25 (1.56–3.24)	

∗ Responder was defined as complete or partial response, and nonresponder as stable or progressive disease.

† Responder was defined as grade III and IV, and nonresponder as grade I and II. ACF indicates adriamycin, cisplatin, and 5-fluorouracil; CI, confidence interval; DCF, docetaxel, cisplatin, and 5-fluorouracil; HR, hazard ratio; LN, lymph node; Lt, lower thorax including esophagogastric junction; Mt, middle thorax; NAC, neoadjuvant chemotherapy; PT, primary tumor; SCC, squamous cell carcinoma; Ut, upper thorax.

We also evaluated the influence of pathological regression grade on survival according to disease site (ie, PT and LNs). The 5-year RFS rates were 78.0% for PT-responders/LNs-responders, 29.4% for PT-responders/LNs-nonresponders, 59.7% for PT-non-responders/LNs-responders, and 26.1% for PT-nonresponders/ LNs-nonresponders (Fig. 3E). To further investigate the impact of pathological LN findings on survival, we divided the patients into 4 groups based on total LN regression grade and ypN status. The 5-year RFS rates were 67.9% among ypN0-1/LNs-responders, 39.0% among ypN0-1/LNs-nonresponders, 55.6% among ypN2-3/LNs-responders, and 16.3% among ypN2-3/LNs-nonresponders (Supplementary Fig. S3A, http://links.lww.com/SLA/C380). We evaluated the relationship of survival impact between total LN regression grade and Union for International Cancer Control pTNM staging system, specifically in pStage II-III diseases. Notably, patients with pStage III disease and LN response had similar or even better 5-year RFS rates compared to patients with pStage II disease who were LN nonresponders (54.2 vs 43.5%, *P* = 0.62) (Supplementary Fig. S3B, http://links.lww.com/SLA/C380).

**FIGURE 4:**
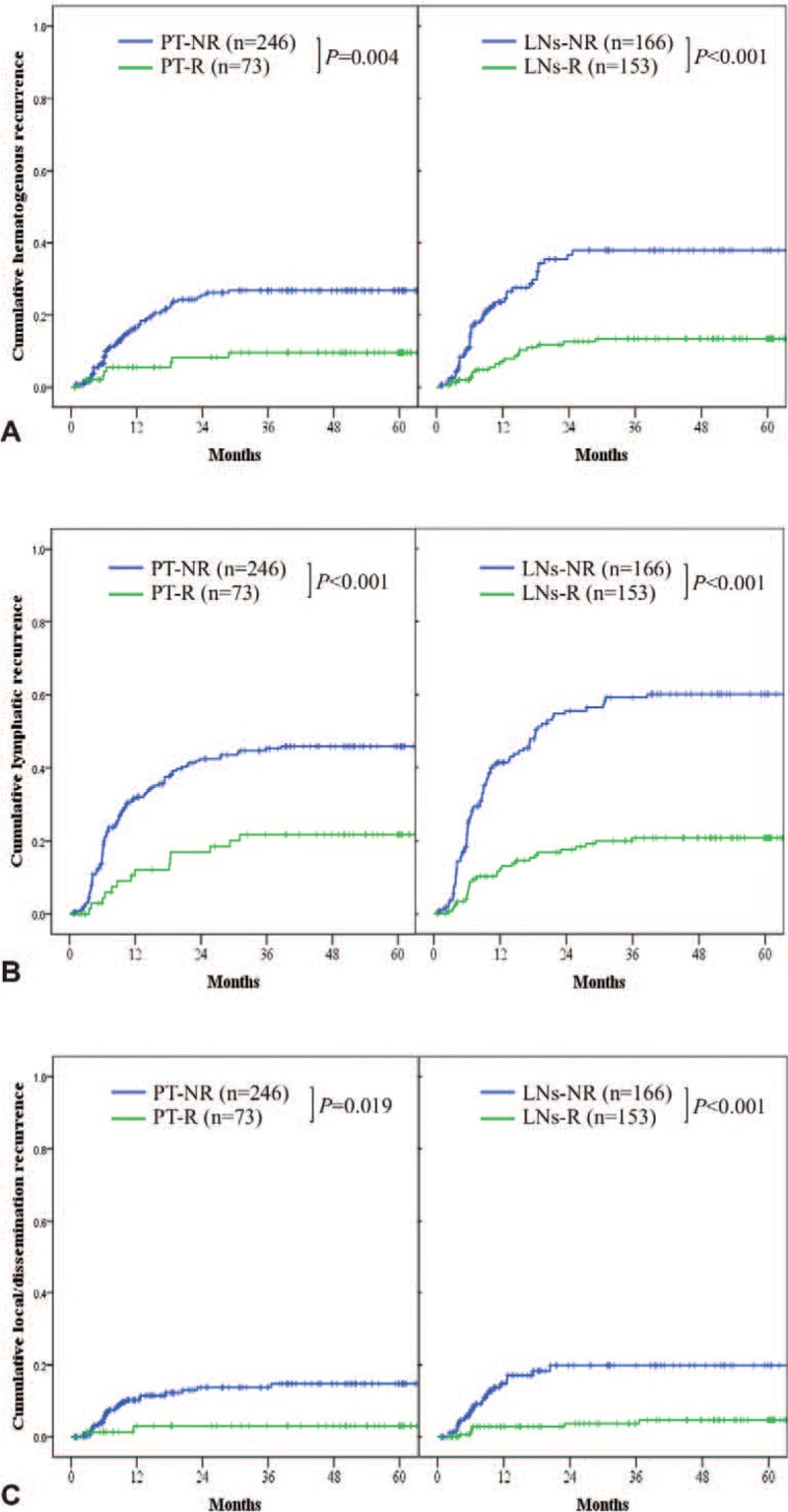
Kaplan-Meier cumulative recurrences, including (A) hematogenous recurrence, (B) lymphatic recurrence, and (C) local or dissemination recurrence, among 319 patients with clinically positive lymph nodes (LNs), according to primary tumor (PT) regression grade and total LN regression grade. NR indicates nonresponders; R, responders.

Among the 319 patients, 151 (47.3%) exhibited recurrences within 5 years after surgery. Figure 4 shows the Kaplan-Meier curves of cumulative recurrences in the 319 patients according to PT and total LN regression grade. Compared with nonresponders, respond-ers in terms of PT regression grade showed significantly lower rates of hematogenous recurrences (29.7% vs 10.7%, *P* = 0.004; Fig. 4A), lymphatic recurrences (45.8% vs 21.8%, *P* < 0.001; Fig. 4B), and local or dissemination recurrences (14.7% vs 3.1%, *P* = 0.019; Fig. 4C). Similarly, compared to nonresponders, the responders in terms of total LN regression grade showed significantly lower rates of hematogenous recurrences (37.9% vs 13.5%, *P* < 0.001; Fig. 4A), lymphatic recurrences (60.2% vs 20.8%, *P* < 0.001; Fig. 4B), and local or dissemination recurrences (19.8% vs 4.8%, *P* < 0.001; Fig. 4C). Notably, these differences were more prominent between groups defined by total LN regression grade than by PT regression grade.

## DISCUSSION

In the present study, we aimed to test a novel system for evaluating the pathological LN response to NAC among EC patients. We performed pathological assessment of all metastatic LNs with and without tumor regression, and nonmetastatic LNs with previous tumor involvement, to determine a total LN regression grade. Among our patients, 23% were PT responders, 48% were responders in terms of total LN regression grade, and 60% showed discordance between the PT and LN regression grades. Total LN regression grade significantly correlated with the number of cN-positive LNs, ypTNM categories, lymphovascular invasion, clinical PT response, and pathological PT regression grade. Moreover, the total LN regression grade, but not pathological PT regression grade, was an independent risk factor for poor RFS. Compared to nonresponders, responders in terms of total LN regression grade exhibited significantly lower rates of hematogenous, lymphatic, and local or dissemination recurrences, and these differences were more prominent than those based on PT response. This study was the first to evaluate total pathological LN response to NAC, and its clinical utility for predicting long-term outcomes, in a large series of EC patients with uniform clinical background.

Intriguingly, our results demonstrated that LN regression grade was a better prognostic factor than PT regression grade. This is consistent with our previous findings that LN clinical response was a better prognostic factor for survival than the PT clinical response.^13^ Two other studies have evaluated pathological LN regression following NAC—one including 256 esophageal adenocarcinoma patients and the other including 110 esophageal squamous cell carcinoma patients—and also demonstrated that LN regression grade was a better prognostic factor for survival than PT regression grade.^27^^,^^28^ In our present study, 60% of patients showed discordance between the PT and LN pathological regression grades, implying the clinical importance of evaluating the response to NAC in both the PT and LNs. Notably, 48.0% of patients exhibited a better response in LNs than PT, whereas only 11.9% showed a better response in PT compared with LNs. This finding could be explained by difference in biological behaviors between PT and LNs, which may influence the therapeutic effects of NAC, possibly in association with tumor size, drug delivery, or immunological microenvironment.^31^ One of the independent prognostic parameters analyzed in our study, pN status, has reportedly shown stronger association with prognosis compared with pT status.^8^^,^^9^ Thus, a combination of ypN status and LN regression grade, indicating the pathological findings of LNs, may be a good indicator for predicting prognosis. Moreover, given that the present LN regression grade further subclassified survival of pStage II-III cases, integration of LN regression grade and pTNM staging might enable more accurate stratification of patient survival, and thus improvement of the indications for adjuvant chemotherapy among EC patients undergoing NAC plus surgery.

A previous study demonstrated greater interobserver agreement with the Becker system compared to the Mandard system when evaluating PT of esophageal adenocarcinoma.^32^ Thus, here we used the Becker system to evaluate the therapeutic effect of NAC on LNs. This system refers to the area of viable tumor and the tumor bed area, and is thus convenient to use in cases of multiple metastatic LNs. In the present study, we validated the grading system by using the software to calculate the sum of the LN area. This revealed high concordance between the gradings conducted by pathologists and the results achieved with the software, even for cases involving large numbers of metastatic LNs. Recent decades have seen rapid development of artificial intelligence technology in the field of digital pathology,^33^ which could help in the evaluation of LN responses using our grading system. Overall, our present system might be reliable with a promising future.

When determining the LN regression grade, the heterogeneity of LN responses within a case becomes an important issue. We found that approximately 40% of cases exhibited a mixed pattern of LN regression (Categories 2–4 in Fig. 2A), suggesting that response grading should include consideration of heterogeneity. Thus, we determined the total LN regression grade to reflect the pathological response of all LNs with present or previous tumor involvement (cN positive before NAC), using the summed viable tumor area and whole tumor bed area. This system enabled evaluation of the real proportion of regression area among all metastatic LNs. In a previous study, Davies et al. determined the best response grade of metastatic LNs for cases exhibiting a mixed pattern of LN responses,^27^ which did not reflect the trend of the whole tumor feature and, thus, could lead to overestimation of tumor response. In another study, Kadota et al calculated the proportion of LNs showing regression among all clinically positive LNs, and categorized patients with ≥50% as responders and those with <50% as nonresponders.^28^ Although this system seemed to reflect the response of all cN-positive LNs, it did not account for the area of all metastatic LNs. In our preliminary study, we investigated the relationship between survival and these previously reported grading systems, and we found that the total LN regression grade exhibited the best correlation with survival (data not shown). Meanwhile, a previous study reported that a small number of dissected LNs was associated with poor survival among patients with EC who underwent preoperative CRT or no preoperative treatment.^34^^,^^35^ However, in these studies, the cut-off value for the number of dissected LNs was ∼20, as opposed to the median number of 57 dissected LNs in our present study (only 4 patients had ≤20 dissected LNs; data not shown), suggesting that the number of dissected LNs in our study was sufficient to support our conclusions.

Our study has several limitations. First, this study had a retrospective design and was performed at a single institution. However, because we collected the data from consecutive NAC patients with EC, and all patients received the same treatment strategy, we believe that selection bias was minimized. Second, it is sometimes challenging to determine the tumor bed area in LNs, especially with a very small area of fibrotic changes, due to limited information. Thus, chronic inflammatory changes could be mistaken for therapeutic changes, leading to overestimation of cN-positive LNs with complete response to NAC (grade IV). Therefore, we investigated the relationship between LN regression grade and the LN sizes before NAC, and found no differences in the initial LN sizes among the 4 categories of LN regression grades (Supplementary Fig. S2, http://links.lww.com/SLA/C379). Moreover, we identified no correlation between LN regression grade and the number of LNs with a tumor bed diameter of <500 μm (data not shown). These findings suggested a low possibility that cN positivity was overestimation in this study. Third, in this study, LN regression grade was evaluated based on only 1 sectioned surface of the LN. In breast cancer, sentinel LNs are entirely sectioned and immunostained to detect micrometastasis.^36^^,^^37^ Our method of evaluating LN regression may have led to overestimation of pathological complete response (grade IV). Finally, this study included only patients who had received NAC using ACF or DCF. The optimized categorization of pathological response evaluation in both PT and LNs, or the difference of recurrence patterns, may change with different chemotherapeutic regimens or CRT. Further studies are needed to investigate this point.

In conclusion, here we demonstrated a novel system for grading pathological LN response, integrating all metastatic LNs with present or previous tumor involvement, among a large series of EC patients who underwent NAC plus surgery. This system showed a greater association with long-term survival and recurrence pattern compared to evaluating PT response, which is a conventional method of pathological response evaluation. Although these findings should be validated in a prospective study of a larger scale, the present information might contribute to optimizing treatment strategies, and to eventually improving survival in patients with metastatic EC.

## Supplementary Material

## References

[1] Bray F Ferlay J Soerjomataram I . Global cancer statistics 2018: GLOBOCAN estimates of incidence and mortality worldwide for 36 cancers in 185 countries. *CA Cancer J Clin* 2018; 68:394–424. 30207593 10.3322/caac.21492

[2] Ando N Kato H Igaki H . A randomized trial comparing postoperative adjuvant chemotherapy with cisplatin and 5-fluorouracil versus preoperative chemotherapy for localized advanced squamous cell carcinoma of the thoracic esophagus (JCOG9907). *Ann Surg Oncol* 2012; 19:68–74. 21879261 10.1245/s10434-011-2049-9

[3] Allum WH Stenning SP Bancewicz J . Long-term results of a randomized trial of surgery with or without preoperative chemotherapy in esophageal cancer. *J Clin Oncol* 2009; 27:5062–5067. 19770374 10.1200/JCO.2009.22.2083

[4] Makino T Miyata H Yamasaki M . Utility of response evaluation to neoadjuvant chemotherapy by (18)F-fluorodeoxyglucose-positron emission tomography in locally advanced esophageal squamous cell carcinoma. *Surgery* 2010; 148:908–918. 20378140 10.1016/j.surg.2010.02.016

[5] Mandard AM Dalibard F Mandard JC . Pathologic assessment of tumor regression after preoperative chemoradiotherapy of esophageal carcinoma. Clinicopathologic correlations. *Cancer* 1994; 73:2680–2686. 8194005 10.1002/1097-0142(19940601)73:11<2680::aid-cncr2820731105>3.0.co;2-c

[6] Becker K Mueller JD Schulmacher C . Histomorphology and grading of regression in gastric carcinoma treated with neoadjuvant chemotherapy. *Cancer* 2003; 98:1521–1530. 14508841 10.1002/cncr.11660

[7] Schneider PM Baldus SE Metzger R . Histomorphologic tumor regression and lymph node metastases determine prognosis following neoadjuvant radiochemotherapy for esophageal cancer: implications for response classification. *Ann Surg* 2005; 242:684–692. 16244542 10.1097/01.sla.0000186170.38348.7b PMC1409844

[8] Doki Y Ishikawa O Takachi K . Association of the primary tumor location with the site of tumor recurrence after curative resection of thoracic esophageal carcinoma. *World J Surg* 2005; 29:700–707. 16078126 10.1007/s00268-005-7596-4

[9] Matsuyama J Doki Y Yasuda T . The effect of neoadjuvant chemotherapy on lymph node micrometastases in squamous cell carcinomas of the thoracic esophagus. *Surgery* 2007; 141:570–580. 17462456 10.1016/j.surg.2006.11.007

[10] Kijima F Natsugoe S Takao S . Detection and clinical significance of lymph node micrometastasis determined by reverse transcription-polymerase chain reaction in patients with esophageal carcinoma. *Oncology* 2000; 58:38–44. 10644939 10.1159/000012077

[11] Yasuda T Yano M Miyata H . Prognostic significance of (18)F-fluoro-deoxyglucose positron emission tomography (FDG-PET)-positive lymph nodes following neoadjuvant chemotherapy and surgery for resectable thoracic esophageal squamous cell carcinoma. *Ann Surg Oncol* 2015; 22:2599–2607. 25524011 10.1245/s10434-014-4299-9

[12] Makino T Doki Y Miyata H . Use of (18)F-fluorodeoxyglucose-positron emission tomography to evaluate responses to neo-adjuvant chemotherapy for primary tumor and lymph node metastasis in esophageal squamous cell carcinoma. *Surgery* 2008; 144:793–802. 19081023 10.1016/j.surg.2008.06.026

[13] Urakawa S Makino T Yamasaki M . Lymph node response to neoadjuvant chemotherapy as an independent prognostic factor in metastatic esophageal cancer. *Ann Surg* 2021; 273:1141–1149. 31274656 10.1097/SLA.0000000000003445

[14] Hagi T Makino T Yamasaki M . Dysphagia score as a predictor of adverse events due to triplet chemotherapy and oncological outcomes in 434 consecutive patients with esophageal cancer. *Ann Surg Oncol* 2019; 26:4754–4764. 31452051 10.1245/s10434-019-07744-7

[15] Hashimoto T Makino T Yamasaki M . The pattern of residual tumor after neoadjuvant chemotherapy for locally advanced esophageal cancer and its clinical significance. *Ann Surg* 2020; 271:875–884. 30829694 10.1097/SLA.0000000000003129

[16] Makino T Yamasaki M Tanaka K . Importance of positron emission tomography for assessing the response of primary and metastatic lesions to induction treatments in T4 esophageal cancer. *Surgery* 2017; 162:836–845. 28711321 10.1016/j.surg.2017.06.007

[17] Makino T Yamasaki M Miyazaki Y . Short- and long-term outcomes of larynx-preserving surgery for cervical esophageal cancer: analysis of 100 consecutive cases. *Ann Surg Oncol* 2016; 23:858–865. 27527716 10.1245/s10434-016-5511-x

[18] Makino T Yamasaki M Tanaka K . Metabolic tumor volume change predicts long-term survival and histological response to preoperative chemotherapy in locally advanced esophageal cancer. *Ann Surg* 2019; 270:1090–1095. 29727327 10.1097/SLA.0000000000002808

[19] Makino T Yamasaki M Miyazaki Y . Utility of initial induction chemotherapy with 5-fluorouracil, cisplatin, and docetaxel (DCF) for T4 esophageal cancer: a propensity score-matched analysis. *Dis Esophagus* 2018; 31:dox130. 10.1093/dote/dox130 29190316

[20] Xxx . Japan Esophageal Society. Japanese classification of esophageal cancer, 11th edition: part I. *Esophagus* 2017; 14:1–36. 28111535 10.1007/s10388-016-0551-7 PMC5222932

[21] Brierley J Gospodarowicz M Wittekind C . TNM Classification of Malignant Tumors. 8th edition Oxford: Wiley-Blackwell; 2017.

[22] Yamasaki M Yasuda T Yano M . Multicenter randomized phase II study of cisplatin and fluorouracil plus docetaxel (DCF) compared with cisplatin and fluorouracil plus Adriamycin (ACF) as preoperative chemotherapy for resectable esophageal squamous cell carcinoma (OGSG1003). *Ann Oncol* 2017; 28:116–120. 27687307 10.1093/annonc/mdw439

[23] Shiraishi O Yamasaki M Makino T . Feasibility of preoperative chemotherapy with docetaxel, cisplatin, and 5-fluorouracil versus adriamycin, cisplatin, and 5-fluorouracil for resectable advanced esophageal cancer. *Oncology* 2017; 92:101–108. 27907921 10.1159/000452765

[24] Makino T Yamasaki M Tanaka K . The impact of prophylactic administration of a neutrophil elastase inhibitor on the postoperative course in older patients undergoing esophagectomy for esophageal cancer: a propensity score-matched analysis. *Esophagus* 2017; 14:241–248.

[25] Makino T Yamasaki M Miyata H . Solitary lymph node recurrence of esophageal squamous cell carcinoma: surgical failure or systemic disease? *Ann Surg Oncol* 2016; 23:2087–2093. 26762271 10.1245/s10434-015-5086-y

[26] Lester SC . Manual of Surgical Pathology. 3rd edition Philadelphia, PA: Elsevier; 2010.

[27] Davies AR Myoteri D Zylstra J . Lymph node regression and survival following neoadjuvant chemotherapy in oesophageal adenocarcinoma. *Br J Surg* 2018; 105:1639–1649. 30047556 10.1002/bjs.10900

[28] Kadota T Hatogai K Yano T . Pathological tumor regression grade of metastatic tumors in lymph node predicts prognosis in esophageal cancer patients. *Cancer Sci* 2018; 109:2046–2055. 29601131 10.1111/cas.13596 PMC5989742

[29] Philippron A Bollschweiler E Kunikata A . Prognostic relevance of lymph node regression after neoadjuvant chemoradiation for esophageal cancer. *Semin Thorac Cardiovasc Surg* 2016; 28:549–558. 28043475 10.1053/j.semtcvs.2016.04.003

[30] Nieman DR Peyre CG Watson TJ . Neoadjuvant treatment response in negative nodes is an important prognosticator after esophagectomy. *Ann Thorac Surg* 2015; 99:277–283. 25442991 10.1016/j.athoracsur.2014.07.037

[31] Wen J Luo KJ Liu QW . The epithelial-mesenchymal transition phenotype of metastatic lymph nodes impacts the prognosis of esophageal squamous cell carcinoma patients. *Oncotarget* 2016; 7:37581–37588. 27147562 10.18632/oncotarget.9036 PMC5122333

[32] Karamitopoulou E Thies S Zlobec I . Assessment of tumor regression of esophageal adenocarcinomas after neoadjuvant chemotherapy: comparison of 2 commonly used scoring approaches. *Am J Surg Pathol* 2014; 38:1551–1556. 25140894 10.1097/PAS.0000000000000255

[33] Bera K Schalper KA Rimm DL . Artificial intelligence in digital pathology - new tools for diagnosis and precision oncology. *Nat Rev Clin Oncol* 2019; 16:703–715. 31399699 10.1038/s41571-019-0252-y PMC6880861

[34] Ho HJ Chen HS Hung WH . Survival impact of total resected lymph nodes in esophageal cancer patients with and without neoadjuvant chemoradiation. *Ann Surg Oncol* 2018; 25:3820–3832. 30284131 10.1245/s10434-018-6785-y

[35] Altorki NK Zhou XK Stiles B . Total number of resected lymph nodes predicts survival in esophageal cancer. *Ann Surg* 2008; 248:221–226. 18650631 10.1097/SLA.0b013e31817bbe59

[36] Protocol for the Examination of Specimens From Patients With Invasive Carcinoma of the Breast. Based on AJCC/UICC TNM, 7th edition. College of American Pathologists: Protocol web posting date: June 2016. https://webapps.cap.org/apps/docs/committees/cancer/cancer_protocols/2012/BreastInvasive_12protocol_3100.pdf#search=’College+of+American+Pathologists.+Protocol+for+the+Examination+of+Speci-mens+AQ10+From+Patients+With+Invasive+Carcinoma+of+the+Breast+Based+on+AJCC%2FUICC+TNM%2C+7th+edition.

[37] Veronesi U Paganelli G Viale G . Sentinel lymph node biopsy and axillary dissection in breast cancer: results in a large series. *J Natl Cancer Inst* 1999; 91:368–373. 10050871 10.1093/jnci/91.4.368

