# Supplementary material for: Pathological Regression of Lymph Nodes Better Predicts Long-term Survival in Esophageal Cancer Patients Undergoing Neoadjuvant Chemotherapy Followed by Surgery
Source: Ann Surg. 2020 Jul 14;275(6):1121–9. doi: 10.1097/SLA.0000000000004238 (PMC10060043; doi:10.1097/SLA.0000000000004238)

**SUPPLEMENTARY FIGURE S1.** Flow chart of patient eligibility for inclusion in the present study. NAC indicates neoadjuvant chemotherapy; ACF, Adriamycin, cisplatin, and 5-fluorouracil; DCF, docetaxel, cisplatin, and 5-fluorouracil; and LNs, lymph nodes.


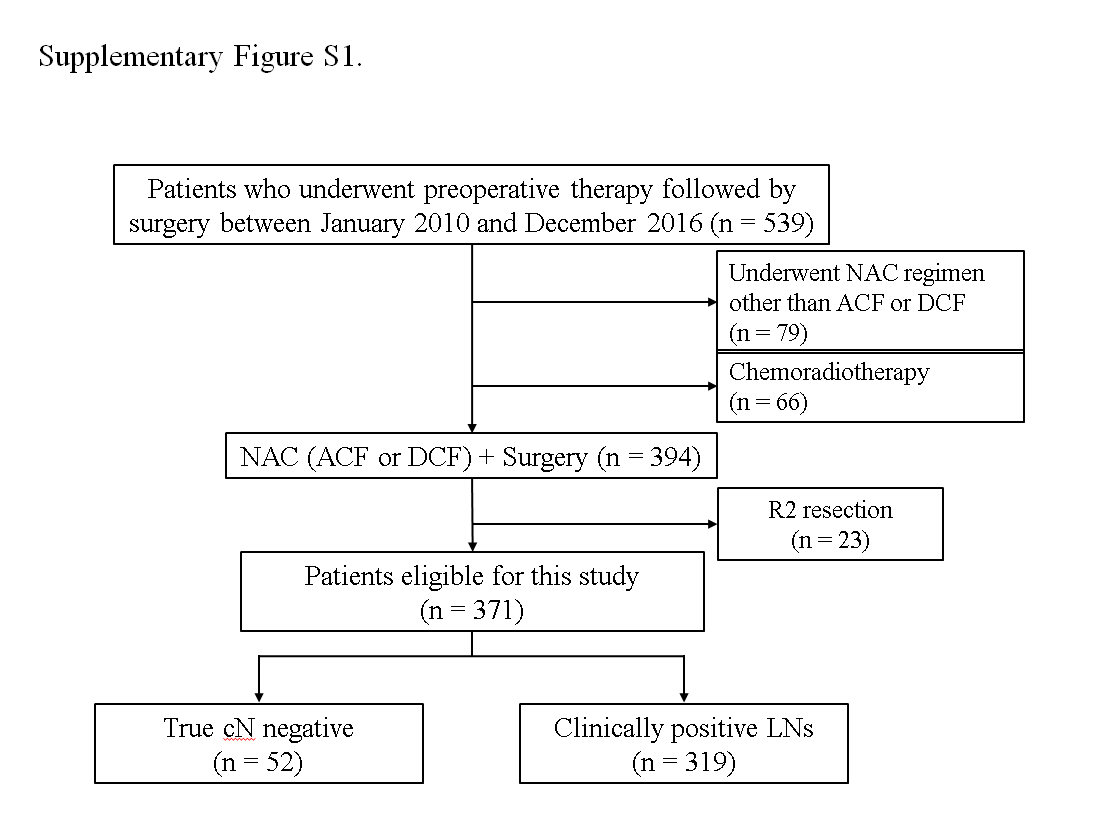

Supplement: Supplementary file 1 [file ansu-275-1121-s001.doc]
