# Supplementary material for: Pathological Regression of Lymph Nodes Better Predicts Long-term Survival in Esophageal Cancer Patients Undergoing Neoadjuvant Chemotherapy Followed by Surgery
Source: Ann Surg. 2020 Jul 14;275(6):1121–9. doi: 10.1097/SLA.0000000000004238 (PMC10060043; doi:10.1097/SLA.0000000000004238)

**SUPPLEMENTARY FIGURE S2.** Relationship between total lymph node regression grade and the summed lengths of minor axes of pretreatment lymph nodes, as measured by pre-therapeutic CT scan. Circle dots indicate the cases. Box plots indicate the levels of the first, second (median), and third quartiles. Bars show the lowest data points within 1.5-fold of the interquartile range (IQR) from the lower quartile boundary, and the highest data points within 1.5-fold of the IQR from the upper quartile boundary. No significant differences were seen between the different grades.


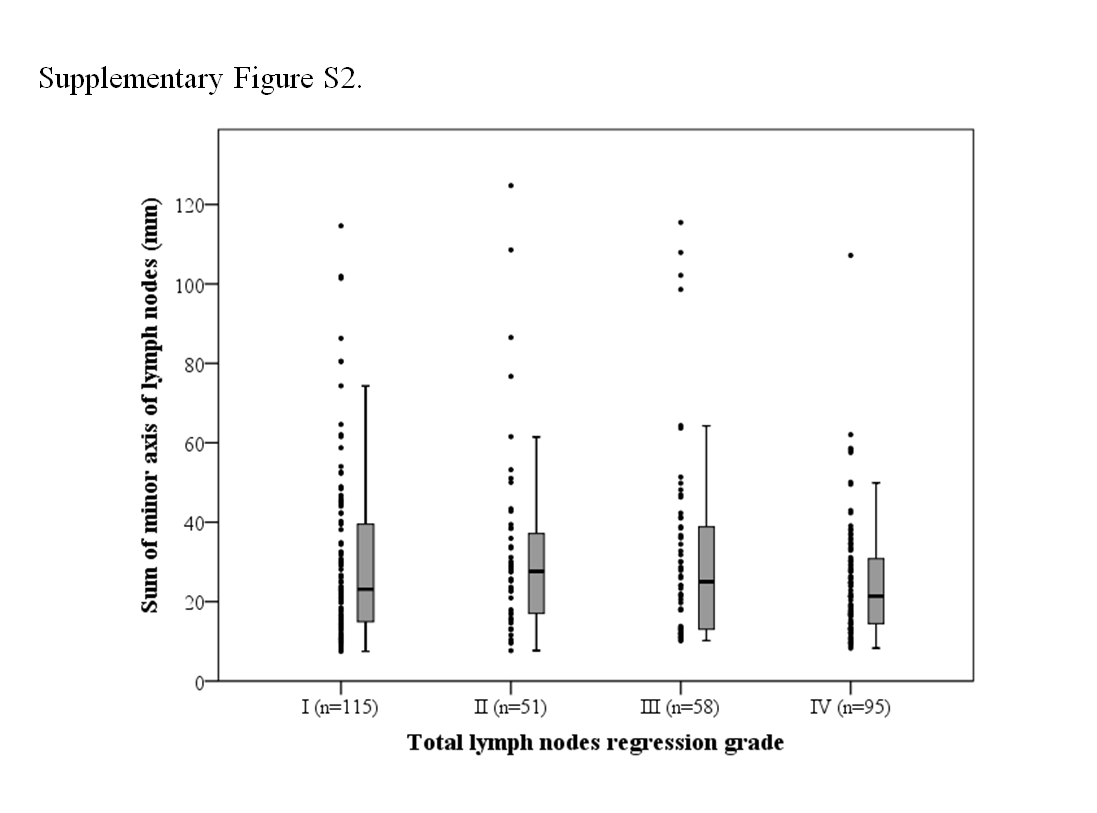

Supplement: Supplementary file 2 [file ansu-275-1121-s002.doc]
