# Supplementary material for: Pathological Regression of Lymph Nodes Better Predicts Long-term Survival in Esophageal Cancer Patients Undergoing Neoadjuvant Chemotherapy Followed by Surgery
Source: Ann Surg. 2020 Jul 14;275(6):1121–9. doi: 10.1097/SLA.0000000000004238 (PMC10060043; doi:10.1097/SLA.0000000000004238)

**SUPPLEMENTARY FIGURE S3.** Kaplan-Meier recurrence-free survival classified into four groups based on total lymph node (LN) regression grade along with (A) parameters of ypN status (319 patients with clinically positive LNs) or (B) pStage based on UICC pTNM staging system (217 patients with pStage II-III). NR indicates non-responders; and R, responders.


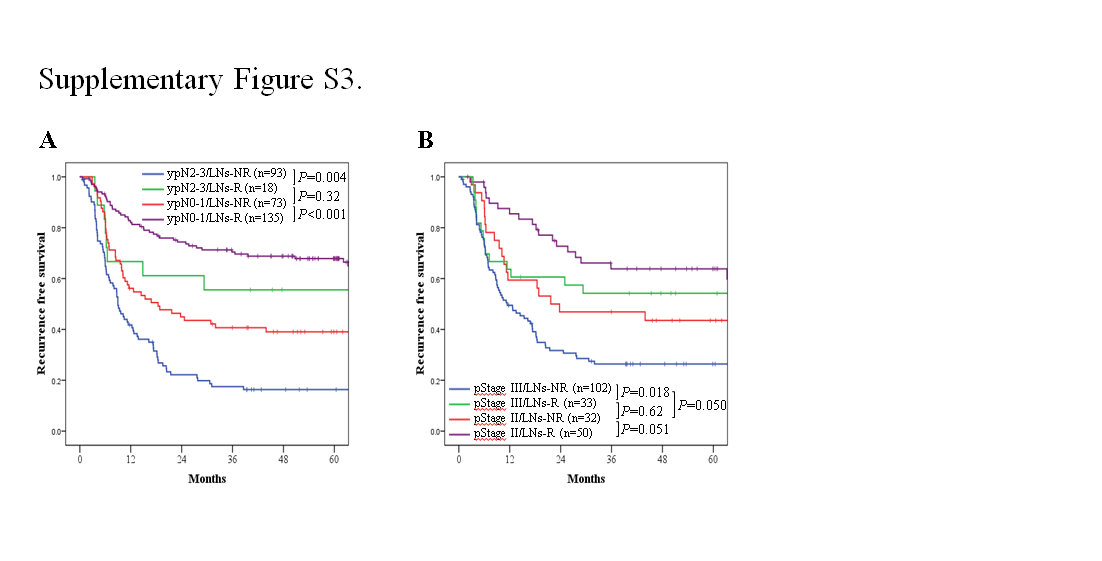

Supplement: Supplementary file 3 [file ansu-275-1121-s003.doc]
