# Supplementary material for: Pathological Regression of Lymph Nodes Better Predicts Long-term Survival in Esophageal Cancer Patients Undergoing Neoadjuvant Chemotherapy Followed by Surgery
Source: Ann Surg. 2020 Jul 14;275(6):1121–9. doi: 10.1097/SLA.0000000000004238 (PMC10060043; doi:10.1097/SLA.0000000000004238)
Supplement: Supplementary file 4 [file ansu-275-1121-s004.docx]

**SUPPLEMENTARY TABLE S1.** Patient Characteristics

|  |  | n = 371 | (%) |
| --- | --- | --- | --- |
| Age in years | Median (range) | 68 (35–83) |  |
| Sex | Male  Female | 318  53 | 85.7  14.3 |
| Location | Ut  Mt  Lt | 65  160  146 | 17.5  43.1  39.4 |
| Histological differentiation (SCC) | Well  Moderate  Poor  Unknown | 102  227  40  2 | 27.5  61.2  10.8  0.5 |
| Residual tumor | R0  R1 | 364  7 | 98.1  1.9 |
| cT | 1  2  3  4 | 19  76  216  60 | 5.1  20.5  58.2  16.2 |
| cN | 0  1  2  3 | 85  243  40  3 | 22.9  65.5  10.8  0.8 |
| cM | 0  1 | 305  66 | 82.2  17.8 |
| cStage | I  II  II  IV | 13  110  182  66 | 3.5  29.6  49.1  17.8 |
| Number of dissected LNs | Median (range) | 57 (14–185) |  |
| Number of clinically positive LNs^a^ | Median (range) | 2 (0–41) |  |
| Summed minor axes of pretreatment LNs, mm | Median (range) | 22.2 (7.2–124.7) |  |
| NAC regimen | FAP  DCF | 107  264 | 28.8  71.2 |
| ypT | 0  1  2  3  4 | 45  95  65  162  4 | 12.1  25.6  17.5  43.7  1.1 |
| ypN | 0  1  2  3 | 145  114  75  37 | 39.1  30.7  20.2  10.0 |
| Number of pathologically positive LNs | Median (range) | 1 (0–40) |  |
| ypM | 0  1 | 332  39 | 89.5  10.5 |
| ypStage | 0  I  II  II  IV | 36  70  87  139  39 | 9.7  18.9  23.4  37.5  10.5 |
| Lymphatic invasion | Negative  Positive  Unknown | 125  219  27 | 33.7  59.0  7.3 |
| Vascular invasion | Negative  Positive  Unknown | 239  105  27 | 64.4  28.3  7.3 |

^a^ Including LNs with evidence of regression or previous tumor involvement.

Ut indicates upper thorax; Mt, middle thorax; Lt, lower thorax including esophagogastric junction; SCC, squamous cell carcinoma; LNs, lymph nodes; NAC, neoadjuvant chemotherapy; ACF, Adriamycin, cisplatin, and 5-fluorouracil; and DCF, docetaxel, cisplatin, and 5-fluorouracil.
